# Rising trends in the burden of migraine and tension-type headache among adolescents and young adults globally, 1990 to 2019

**DOI:** 10.1186/s10194-023-01634-w

**Published:** 2023-07-27

**Authors:** Ying Yang, Yu Cao

**Affiliations:** Xiang’an Hospital of Xiamen University, Xiamen, China

**Keywords:** Headache disorders, Migraine, Tension-type headache, Global trends, Global Burden of Disease Study, Joinpoint regression

## Abstract

**Background:**

Headache disorders are major contributors to disability in adolescents and young adults worldwide. We aimed to analyze global trends in the burden of migraine and tension-type headache in 10 to 24-year-olds from 1990 to 2019.

**Methods:**

Data were obtained from the Global Burden of Disease Study (GBD) 2019 to examine trends in incidence, prevalence, and disability-adjusted life years (DALYs) for migraine and tension-type headache in adolescents and young adults by location, age, sex and sociodemographic index (SDI). Joinpoint regression analyzed trends and identified years of significant change.

**Results:**

Globally, migraine and tension-type headache incidence, prevalence, and DALYs increased from 1990 to 2019, though with some fluctuations. The highest growth in migraine incidence occurred in males and individuals aged 10-14, while for tension-type headache incidence, it was observed in males and individuals aged 20-24. Prevalence and DALYs were highest for both disorders in females and those aged 20–24 years. Incidence increased fastest in East Asia for migraine and tension-type headache. For migraine, Tropical Latin America had the fastest increase in prevalence and DALYs. East Asia had the fastest increase in prevalence of tension-type headache, while North Africa and the Middle East had the highest growth in DALYs. Some high-income countries such as Singapore, Norway, and Iran (Islamic Republic of) demonstrated fast increases, while a few countries including Qatar, Thailand and Ethiopia decreased.

**Conclusions:**

The incidence, prevalence and disability from migraine and tension-type headache are rising in adolescents and young adults, especially in males, older youth and middle SDI populations. The increasing trends highlight the need for targeted interventions focused on prevention and control in priority populations. Continued monitoring of trends can help identify strategies to promote headache health and reduce the life-course impacts of headache disorders globally.

**Supplementary Information:**

The online version contains supplementary material available at 10.1186/s10194-023-01634-w.

## Introduction

Headache disorders are among the most common and disabling conditions of the nervous system, affecting almost half of the adult population worldwide [1]. In 2019, headache disorders constituted the second predominant contributor to disability-adjusted life-years (DALYs) in adolescents and young adults aged 10 to 24 years [2]. Recent analyses ranked headache disorders third among 369 diseases/injuries in disability, as measured by years lived with disability (YLDs), across populations and foremost among individuals aged 15 to 49 years, constituting 8% of YLDs from any cause while migraine alone ranked second, responsible for 7.3% of all-cause YLDs [2, 3]. The coexistence of various medical conditions, which represents a facet of the multidimensional and dispersed burden imposed by headache disorders, is prone to result in an elevation of headache-associated disability and cost to communities [4, 5]. They can cause significant personal and societal burden, such as reduced quality of life, impaired school and work performance, increased health care costs, and increased risk of other chronic diseases [6, 7]. Moreover, headache disorders in adolescence may persist or worsen into adulthood, leading to chronic and refractory conditions that impose a high personal and societal cost [8].

However, less attention has been paid to the burden of headache disorders in adolescents and young adults aged 10–24 years [9, 10], who represent a significant proportion of the global population and are at a critical stage of development and transition. Studies describe characteristics of various headache types in youth and young adults, including migraine, tension-type and posttraumatic headache, elucidating their prevalence [11]. These studies have limitations in terms of representativeness, comparability, and generalizability. Moreover, most of these studies were conducted before 2015, and may not reflect the current situation of headache disorders in adolescents and young adults. Therefore, there is a need for a comprehensive and updated assessment of the global, regional and national burdens of headache disorders in adolescents and young adults, using standardized and comparable methods and data sources.

This study aimed to fill this gap by conducting a trend analysis based on the Global Burden of Disease Study (GBD) 2019, which is a systematic and comprehensive effort to measure the health status of populations across 204 countries and territories from 1990 to 2019. The objectives were fourfold: 1) evaluate global trends in migraine and tension-type headache incidence, prevalence, and DALYs by decade from 1990 through 2019; 2) identify years of greatest change; 3) compare trends by age, sex, and sociodemographic index (SDI); and 4) explore regional/national trends and implications for policy/practice.

## Methods

### Study population and data collection

We analyzed data from GBD 2019 to assess global disease burden from 1990 to 2019. The GBD 2019 database includes 369 diseases and injuries spanning 204 countries and territories [12]. For this analysis, we extracted data on migraine and tension-type headache. The diagnoses of migraine and tension-type headache were defined based on the reference case definitions satisfying the International Classification of Headache Disorders-3 (ICHD-3) [13], as specified by the GBD [14].

GBD 2019 provided data on incidence rates, prevalence rates, DALYs rates, incident cases, and prevalent cases. Rates were age-standardized and expressed per 100,000 population. To calculate the 95% uncertainty intervals (UIs), the GBD generated 1000 estimates for each value using their analytical modeling approach. The values at the 25th and 975th rank positions among these 1000 ordered estimates were then utilized to define the lower and upper bounds, respectively, of the 95% UIs. The methodological approach employed in GBD 2019 has been documented in prior related literature [12].

### Sociodemographic index

GBD 2019 produced an index called SDI for each nation. This index captures sociocultural and macroeconomic factors that impact health outcomes in different locations across the world. The SDI incorporates three measures—absolute fertility rate (< 25 years), mean education (≥ 15 years), and income adjusted for time it represents their geometric average [12]. The SDI stratifies countries into quintiles ranging from low to high: low, low-middle, middle, high-middle, high.

### Statistical analysis

The overarching aims were fourfold:1) assess global migraine/tension-type headache incidence prevalence and DALYs trends 1990–2019;2) pinpoint years of greatest change via Joinpoint regression;3) stratify trends by age, sex and SDI;4) examine regional and national trends using average annual percent change (AAPC).

Joinpoint regression analysis [15] was employed to analyse temporal trends in the incidence, prevalence and DALYs of migraine and tension-type headache globally, regionally and nationally. The overall trend was partitioned into multiple periods based upon the identification of joinpoints, which demarcated significant shifts in tendency. AAPCs and 95% confidence intervals (CIs) were calculated for selected time periods to evaluate trends. Moreover, the AAPC was derived as a weighted mean of the annual percentage change (APC) across predefined fixed intervals to comprehensively summarize trends. The weighted Bayesian information criterion were employed through the Joinpoint program to ascertain the optimal model.

The following software were employed in the analyses: R version 4.3.0 and Joinpoint Regression Program version 5.0.2 [15]. Statistical significance was defined as p < 0.05.

## Results

### Global trends

**Fig. 1:**
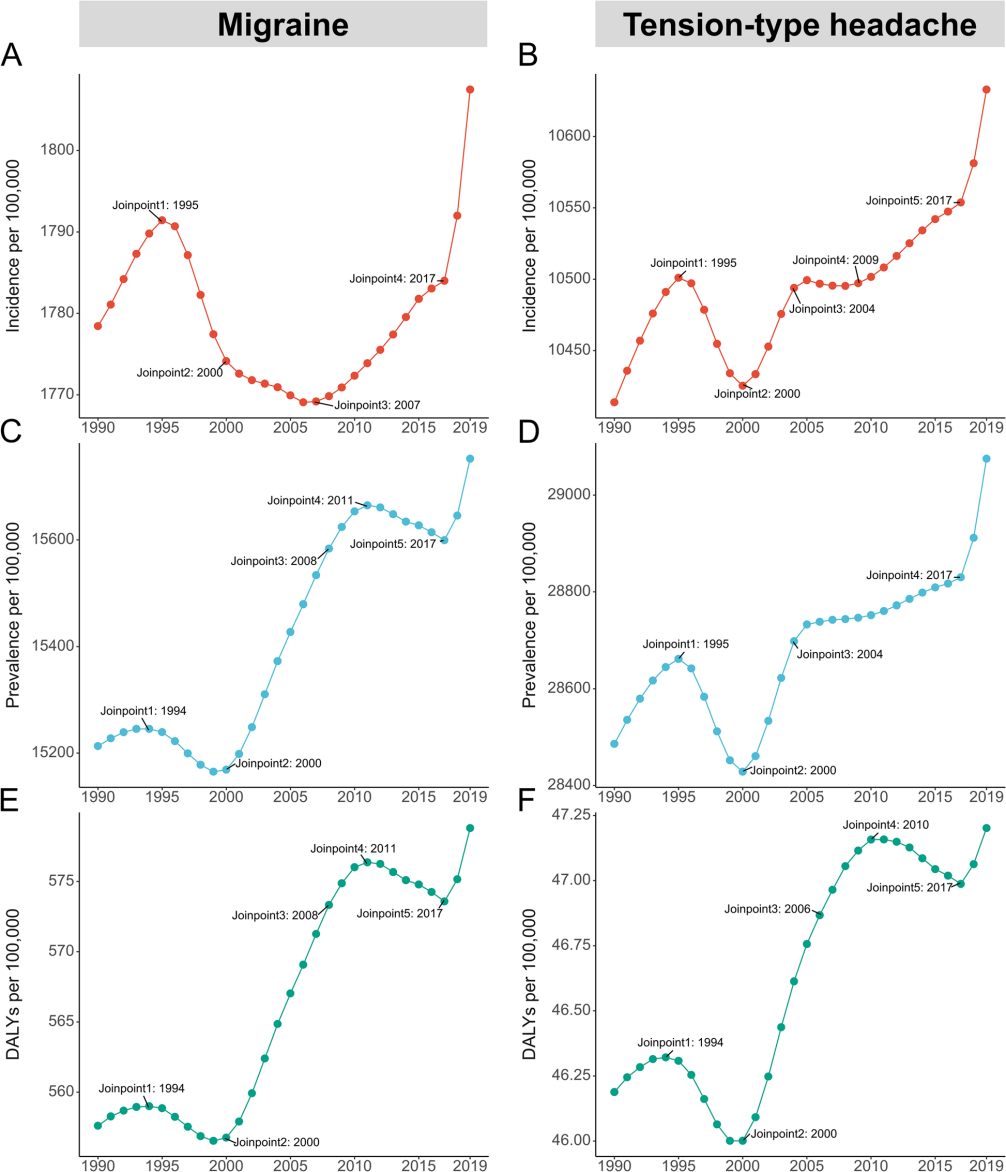
Joinpoint regression analysis for incidence of migraine (**A**), prevalence of migraine (**C**), DALYs of migraine (**E**) and incidence of tension-type headache (**B**), prevalence of tension-type headache (**D**), and DALYs of tension-type headache (**F**) in adolescents and young adults aged 10–24 years from 1990 to 2019. DALYs = disability-adjusted life-years

**Fig. 2:**
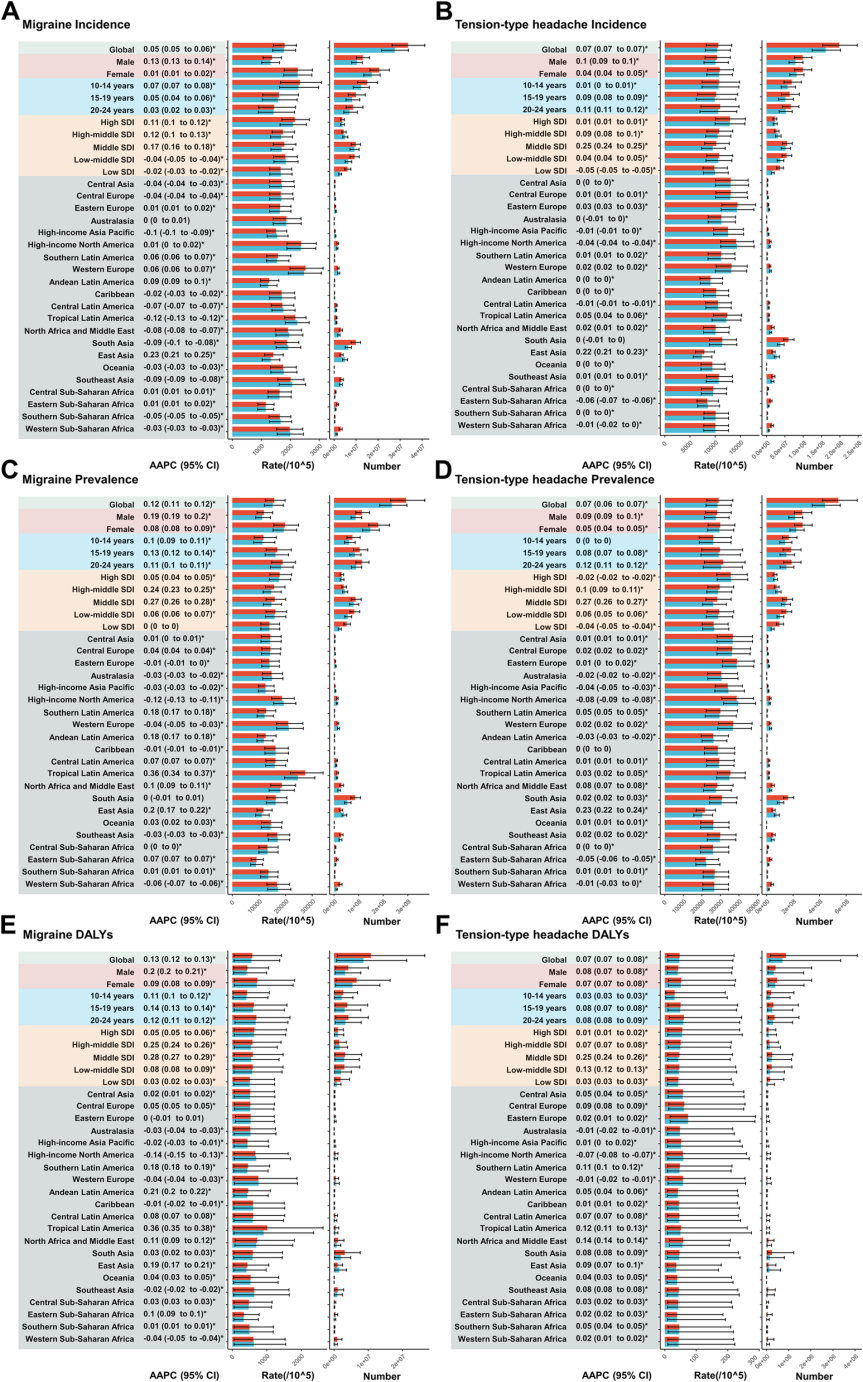
The incidence, prevalence and DALYs of migraine and tension-type headache and their AAPCs from 1990 to 2019 at the global and regional levels. **A** incidence of migraine; **B** incidence of tension-type headache; **C** prevalence of migraine; **D** prevalence of tension-type headache; **E** DALYs of migraine; **F** DALYs of tension-type headache. Data in parentheses are 95% CIs for AAPCs. Blue represents the 1990 data and red represents the 2019 data, with error bars indicating the 95% uncertainty intervals. DALYs = disability-adjusted life-years. UI = uncertainty interval. AAPC = average annual percentage change

Globally, migraine incidence showed an overall increasing trend (AAPC = 0.05; 95% CI: 0.05 to 0.06, Figs. 1, 2 and Table S1) between 1990 and 2019. There was no statistically significant change in migraine incidence from 1990 to 1999 (Table 1). Incidence decreased from 2000 to 2009 (AAPC = -0.02; 95% CI: -0.03 to -0.01, Fig. 1 and Table 1) but increased from 2010 to 2019 (AAPC = 0.21; 95% CI: 0.18 to 0.23, Fig. 1 and Table 1). Joinpoint analysis identified four significant changes in incidence (in 1995, 2000, 2007 and 2017, Fig. 1). Migraine prevalence and DALYs increased from 1990 to 2019 overall, though not consistently. From 1990 to 1999, rates of prevalence and DALYs declined, but they experienced an increase from 2000 to 2009 and from 2010 to 2019 (Fig. 1 and Table 1). Joinpoint analysis found five identical joinpoints for prevalence and DALYs (in 1994, 2000, 2008, 2011 and 2017, Fig. 1).

**Table 1:** Global AAPCs in prevalence, incidence, and DALYs of migraine and tension-type headache

period	Incidence	Prevalence	DALYs
Migraine			
1990–99	0 (-0.01 to 0.01)	-0.04 (-0.05 to -0.03)*	-0.03 (-0.03 to -0.02)*
2000–09	-0.02 (-0.03 to -0.01)*	0.35 (0.33 to 0.36)*	0.38 (0.36 to 0.39)*
2010–19	0.21 (0.18 to 0.23)*	0.05 (0.02 to 0.07)*	0.03 (0.01 to 0.05)*
1990–2019	0.05 (0.05 to 0.06)*	0.12 (0.11 to 0.12)*	0.13 (0.12 to 0.13)*
Tension-type headache			
1990–99	0.02 (0.01 to 0.04)*	-0.01 (-0.03 to 0)*	-0.05 (-0.07 to -0.04)*
2000–09	0.08 (0.07 to 0.09)*	0.13 (0.11 to 0.15)*	0.28 (0.27 to 0.3)*
2010–19	0.13 (0.12 to 0.14)*	0.12 (0.1 to 0.13)*	0 (-0.02 to 0.01)
1990–2019	0.07 (0.07 to 0.07)*	0.07 (0.06 to 0.07)*	0.07 (0.07 to 0.08)*

*AAPC* Average annual percentage change, *DALYs* Disability-adjusted life-years

* indicates the AAPC is significantly different from zero at alpha = 0.05 level

Globally, tension-type headache incidence exhibited an overall upward trend (AAPC = 0.07; 95% CI: 0.07 to 0.07, Figs. 1, 2 and Table 1) from 1990 to 2019. Incidence increased from 1990 to 1999 (AAPC = 0.02; 95% CI: 0.01 to 0.04, Fig. 1, and Table 1), from 2000 to 2009 (AAPC = 0.08; 95% CI: 0.07 to 0.09, Fig. 1 and Table 1), and from 2010 to 2019 (AAPC = 0.13; 95% CI: 0.12 to 0.14, Fig. 1 and Table 1), with an accelerating rate of increase over time. Joinpoint analysis identified five significant changes in incidence (in 1995, 2000, 2004, 2009 and 2017, Fig. 1). The prevalence rate of tension-type headache decreased from 1990 to 1999 but increased from 2000 to 2009 and from 2010 to 2019 (Fig. 1 and Table 1). The overall trend was increasing (Fig. 1 and Table 1). The DALYs rate decreased from 1990 to 1999, increased from 2000 to 2009, and showed no statistically significant change from 2010 to 2019 (Fig. 1 and Table 1). The overall trend was increasing (Figs. 1and Table 1). Joinpoint analysis identified four significant changes in prevalence rate (in 1995, 2000, 2004 and 2017, Fig. 1) and five changes in DALYs rate (in 1994, 2000, 2006, 2010 and 2017, Fig. 1).

### Global trends by sex

The incidence of migraine increased in males (AAPC = 0.13; 95% CI: 0.13 to 0.14, Fig. 2 and Table S1) and females (AAPC = 0.01; 95% CI: 0.01 to 0.02, Fig. 2 and Table S1), faster in males. However, the 2019 incidence rate (2262.77 per 100,000; 95% UI: 1790.26 to 2763.62, Fig. 2 and Table S1) and cases (20,554,715.15; 95% UI: 16,262,528.55 to 25,104,407.96, Fig. 2 and Table S1) were higher in females, as in 1990 (Fig. 2 and Table S1). Prevalence rate and DALYs rate increased in both sexes, faster in males, but females had higher prevalence rates, cases, DALYs rates, and DALYs cases than males in both 2019 and 1990 (Fig. 2 and Table S1).

For tension-type headache, incidence, prevalence and DALYs trends were similar to migraine. Incidence, prevalence and DALYs rate increased in both sexes, faster in males (AAPC > 0, Fig. 2 and Table S1), but females had higher rates and cases of incidence, prevalence, and DALYs in both 2019 and 1990 (Fig. 2 and Table S1).

### Global trends by age group

For all 3 age groups, the migraine incidence increased (AAPC > 0, Fig. 2 and Table S1) from 1990 to 2019, fastest in 10–14-year-olds (AAPC = 0.07; 95% CI: 0.07 to 0.08, Fig. 2 and Table S1). In 2019, this 10–14-year-olds had the highest incidence rate and number of cases (Fig. 2 and Table S1). The prevalence rate and DALYs rate also increased (AAPC > 0, Fig. 2 and Table S1), fastest in 15–19-year-olds (Fig. 2 and Table S1). In 2019, the 20–24-year-olds had the highest prevalence rate, prevalence cases, DALYs rate, and DALYs cases (Fig. 2 and Table S1).

For all 3 age groups, the tension-type headache incidence increased (Fig. 2 and Table S1), similar to migraine. The incidence grew fastest in 20–24-year-olds (AAPC = 0.11; 95% CI: 0.11 to 0.12, Fig. 2 and Table S1). The highest 2019 incidence rate was in 20–24 years (10,989.32 per 100,000; 95% UI: 7209.02 to 14,718.37, Fig. 2 and Table S1); the highest number of incident cases was in 10–14-year-olds (68,780,685.06; 95% UI: 47,537,314.05 to 95,191,441.84, Fig. 2 and Table S1). Excluding 10–14- year-olds where there was no statistically significant change, the prevalence rate and DALYs rate increased in all three age groups. (AAPC > 0, Fig. 2 and Table S1). The prevalence rate grew fastest in 20–24-year-olds (Fig. 2 and Table S1); the DALYs rate grew fastest in 15–19- and 20–24-year-olds (with the same AAPC) (Fig. 2 and Table S1). In 2019, the 20–24-year-olds had the highest prevalence rate, prevalence cases, DALYs rate, and DALYs cases (Fig. 2 and Table S1).

### Global trends by SDI

According to the SDI stratification, the incidence of migraine among the global population aged 10–24 years from 1990 to 2019 decreased in the low-middle SDI and low SDI populations (AAPC < 0, Fig. 2 and Table S1), while it increased in the other three (AAPC > 0, Fig. 2 and Table S1). In 2019, the high SDI population had the highest incidence rate (2163.31per 100,000;95%UI: 1713.11 to 2661.37, Fig. 2 and Table S1), the middle SDI population had the most incident cases (9,876,051.28 per 100,000;95%UI: 7,760,365.78 to 12,071,317.68, Fig. 2 and Table S1), and the middle SDI population grew fastest (AAPC = 0.17; 95%CI: 0.16 to 0.18, Fig. 2 and Table S1).For prevalence rate, except for the low SDI population with no statistical significance, the other four all increased (AAPC > 0, Fig. 2 and Table S1). The middle SDI population saw the fastest growth (Fig. 2 and Table S1), while the high SDI population had the highest prevalence rate (Fig. 2 and Table S1) in 2019. For DALYs rate, all five SDI populations increased (AAPC > 0, Fig. 2 and Table S1). The fastest growth was in the middle SDI population (Fig. 2 and Table S1), and the highest DALYs rate was in the high SDI population (Fig. 2 and Table S1) in 2019.

According to the SDI stratification, incidence of tension-type headache decreased in the low SDI population (AAPC < 0, Fig. 2 and Table S1) but increased in the other four (AAPC > 0, Fig. 2 and Table S1). The fastest growth occurred in the Middle SDI population (AAPC = 0.25; 95%CI: 0.24 to 0.25, Fig. 2 and Table S1), much faster than the second fastest High-middle SDI (AAPC = 0.09; 95%CI: 0.08 to 0.1, Fig. 2 and Table S1). In 2019, The highest incidence rate was in the High SDI population (12,945.16per 100,000; 95%UI: 10,040.77 to 15,993.52, Fig. 2 and Table S1), most incident cases were in the Middle SDI population (56,259,290.14;95%UI: 43,536,222.73 to 70,285,829.34, Fig. 2 and Table S1). The prevalence rate in the High-middle SDI, Middle SDI and Low-middle SDI populations increased (AAPC > 0, Fig. 2 and Table S1), while High SDI and Low SDI decreased (AAPC < 0, Fig. 2 and Table S1). The Middle SDI population had the fastest growth in prevalence rate (Fig. 2 and Table S1), much faster than the second fastest Low-middle SDI (Fig. 2 and Table S1). The highest prevalence rate was in the High SDI population (Fig. 2 and Table S1) in 2019. The DALYs rate increased in all five SDI populations (AAPC > 0, Fig. 2 and Table S1). The Middle SDI population had the fastest growth in DALYs rate (Fig. 2 and Table S1), much faster than other SDI levels (Fig. 2 and Table S1). The highest DALYs rate was in the High SDI population (Fig. 2 and Table S1) in 2019.

### Region trends

From 1990 to 2019, the incidence of migraine among 10–24-year-olds varied by region. The fastest increase was in East Asia (AAPC = 0.23; 95% CI: 0.21 to 0.25, Fig. 2 and Table S1) while the largest decrease was in Tropical Latin America (AAPC = -0.12; 95% CI: -0.13 to-0.12, Fig. 2 and Table S1). In 2019, the highest incidence rate was in Western Europe (2529.64 per 100,000; 95% UI:1993.13 to 3133.21, Fig. 2 and Table S1) and the lowest was in Eastern Sub-Saharan Africa (1155.48 per 100,000; 95% UI: 878.8 to 1437.4, Fig. 2 and Table S1). This was the same as in 1990.Prevalence and DALYs increased the most in Tropical Latin America (Fig. 2 and Table S1) while the largest decrease was in High-income North America (Fig. 2 and Table S1). In 2019, the highest prevalence rate and DALYs rate were in Tropical Latin America (Fig. 2 and Table S1).

From 1990 to 2019, the incidence of tension-type headache in 10–24-year-olds varied by region. The fastest increase in incidence was observed in East Asia (AAPC = 0.22; 95% CI: 0.21 to 0.23, Fig. 2 and Table S1), followed by Tropical Latin America (AAPC = 0.05; 95% CI: 0.04 to 0.06, Fig. 2 and Table S1), with East Asia increasing much faster than Tropical Latin America. The steepest drop was in Eastern Sub-Saharan Africa (AAPC = -0.06; 95% CI -0.07 to -0.06, Fig. 2 and Table S1). In 2019, the highest incidence rate was in Eastern Europe (14,445.72 per 100,000; 95% UI: 11,316.87 to 17,991.69, Fig. 2 and Table S1) and the lowest in East Asia (7871.93 per 100,000; 95% UI: 6077.30 to 9830.16, Fig. 2 and Table S1). South Asia had the most incident cases in 2019 (59,885,006.4, 95% UI: 46,581,368.1 to 74,796,508.9, Fig. 2 and Table S1). The fastest rise in prevalence was in East Asia (Fig. 2 and Table S1) while the fastest drop was in High-income North America (Fig. 2 and Table S1). The increase in DALYs rate was highest in North Africa and the Middle East (Fig. 2 and Table S1) and the steepest decrease in High-income North America (Fig. 2 and Table S1). Eastern Europe (Fig. 2 and Table S1) had the highest prevalence and DALYs, like the highest incidence.

### National trends

**Fig. 3:**
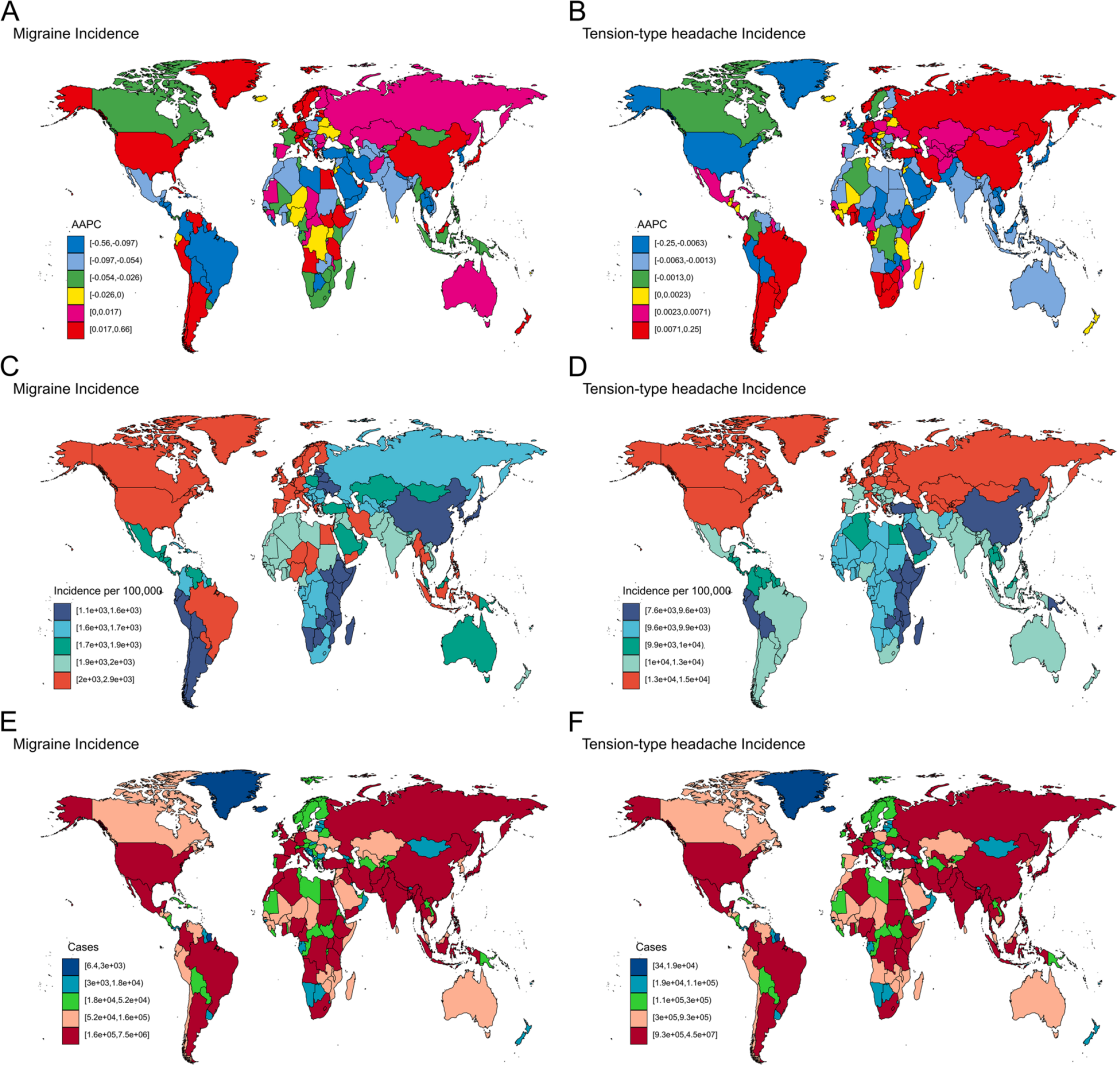
Global map of AAPC for incidence from 1990 to 2019 of migraine (**A**) and tension-type headache (**B**), incidence in 2019 of migraine (**C**) and tension-type headache (**D**) and incidence cases in 2019 of migraine (**E**) and tension-type headache (**F**). AAPC = average annual percentage change

Globally, the incidence trend of migraine among 10–24-year-olds from 1990 to 2019 varied by nation. The fastest increase in incidence was observed in Singapore (AAPC = 0.66; 95% CI: 0.62 to 0.69, Fig. 3 and Table S2), and the fastest decrease was in Qatar (AAPC = -0.56; 95% CI: -0.59 to -0.54, Fig. 3 and Table S2). In 2019, Italy had the highest migraine incidence rate (2853.68 per 100,000; 95% UI: 2290.44 to 3434.40, Fig. 3 and Table S2) and Ethiopia had the lowest (1078.18 per 100,000; 95% UI: 837.33 to 1327.48, Fig. 3 and Table S2). The highest incidence cases in 2019 was observed in India (7,493,754.26, 95% UI: 5,854,508.41 to 9,144,678.87, Fig. 3 and Table S2). Norway had the fastest increase in prevalence rate and DALYs rate (Figure S1, Figure S2 and Table S2), and Thailand had the fastest decrease (Figure S1, Figure S2 and Table S2).

Globally, the incidence trend of tension-type headache among 10–24-year-olds from 1990 to 2019 also varied by nation. The fastest increase in incidence was in Iran (Islamic Republic of) (AAPC = 0.25; 95% CI: 0.22 to 0.26, Fig. 3 and Table S2), and the fastest decrease was in Ethiopia (AAPC = -0.25; 95% CI: -0.26 to -0.24, Fig. 3 and Table S2). In 2019, Ukraine had the highest tension-type headache incidence rate (14,646.71 per 100,000; 95% UI: 11,398.85 to 18,116.71, Fig. 3 and Table S2), and Ethiopia had the lowest (7558.42 per 100,000; 95% UI: 5694.83 to 9808.73, Fig. 3 and Table S2). The highest incidence cases in 2019 was observed in India (45,306,492.4, 95% UI: 35,278,454.6 to 56,578,610.7, Fig. 3 and Table S2). Iran (Islamic Republic of) had the highest increase in both the prevalence rate and DALYs rate, while Ethiopia had the fastest decline in the prevalence rate and Monaco had the fastest decline in DALYs rate (Figure S1, Figure S2 and Table S2).

## Discussion

This analysis offers a comprehensive evaluation of global, regional and national trends in burden of the two most common headache disorders, migraine and tension-type headache, in 10- to 24-year-olds over the past three decades. Our results show that globally, the incidence, prevalence and DALYs of migraine and tension-type headache are increasing in this age group. The increasing trends are more prominent in males, older age groups (15–19 and 20–24 years) and middle SDI populations. Regionally, the burden is increasing fastest in East Asia, North Africa and the Middle East and Tropical Latin America. Some high-income countries like Singapore, Norway and Iran (Islamic Republic of) also show fast increasing trends. However, a few countries such as Qatar, Thailand, Monaco and Ethiopia show decreasing trends.

The increasing global burden of headache disorders in adolescents and young adults highlights the need to strengthen prevention and control efforts targeting this age group. Possible reasons for the increasing trends include lifestyle changes, increasing use of electronic devices, sleep deprivation, and psychological stress in youth [16–20]. The transition to adulthood and new responsibilities during this developmental stage may also contribute to increased stress and headache triggers [21, 22]. The higher burden in males and older age groups suggests the need for targeted interventions focusing on risk factors more prevalent in these populations.

Regionally, the particularly fast increasing trends in East Asia, Latin America and parts of Eastern Europe could reflect rapid societal changes in these regions that influence headache development in youth, such as urbanization, healthcare, and air quality [23–27]. The decreasing trends in a few countries may indicate the success of health promotion and public health efforts in these areas to reduce headache triggers and improve disease management. Continued monitoring of trends in these countries could help identify best practices for headache prevention and control globally.

This study has some limitations. Due to data availability, we were only able to assess trends for two major headache types, migraine and tension-type headache. The trends of other headache disorders remain unknown. In addition, the indicators were modeled at national levels, so subnational variations in trends could not be examined. Furthermore, variations in diagnostic criteria for migraine and tension-type headache can result in differences in reported incidence rates. These variations are influenced by cultural and regional factors that affect case identification. When comparing rates between countries, it is important to exercise caution due to these variations. Implementing standardized diagnostic criteria is essential for accurate comparisons and a comprehensive understanding of the global burden of these conditions. Despite these limitations, this study provides an update and comprehensive assessment of trends in the burden of headache disorders among adolescents and young adults around the world. The results highlight populations that should be prioritized in global efforts to reduce the burden of headache and promote headache health in youth.

In conclusion, this study reveals increasing global trends in the incidence, prevalence and DALYs associated with migraine and tension-type headache among adolescents and young adults over the past 30 years. Targeted strategies are urgently warranted to mitigate headache burden in this at-risk population. Continued monitoring of trends and investigation of underlying causes can help guide global health policy and practice. Reducing the impact of headache disorders in youth has the potential to decrease suffering and improve health, education and economic outcomes over the life course.

## Supplementary Information

**Additional file 1. **

**Additional file 2. **

## Data Availability

Data used in this study can be found on GBD: https://www.healthdata.org/gbd.

## Abbreviations

AAPC: Average annual percent change
DALY: Disability-adjusted life years
APC: Annual percent change
SDI: Sociodemographic Index
CI: Confidence interval
UI: Uncertainty interval
GBD: Global Burden of Disease Study

## References

[1] Headache disorders. https://www.who.int/news-room/fact-sheets/detail/headache-disorders. Accessed 7 June 2023

[2] Vos T, Lim SS, Abbafati C (2020). Global burden of 369 diseases and injuries in 204 countries and territories, 1990–2019: a systematic analysis for the Global Burden of Disease Study 2019. Lancet.

[3] Steiner TJ, Stovner LJ, Jensen R et al (2020) Migraine remains second among the world’s causes of disability, and first among young women: findings from GBD2019. J Headache Pain 21:137. 10.1186/s10194-020-01208-0 10.1186/s10194-020-01208-0 PMC7708887 33267788

[4] Leonardi M, Raggi A (2019) A narrative review on the burden of migraine: when the burden is the impact on people’s life. J Headache Pain 20:41. 10.1186/s10194-019-0993-0 10.1186/s10194-019-0993-0 PMC6734273 31023226

[5] Jensen R, Stovner LJ (2008) Epidemiology and comorbidity of headache. Lancet Neurol 7:354–361. 10.1016/S1474-4422(08)70062-0 10.1016/S1474-4422(08)70062-0 18339350

[6] Brzoska P (2020) Assessment of quality of life in individuals with chronic headache. Psychometric properties of the WHOQOL-BREF. BMC Neurol 20:267. 10.1186/s12883-020-01845-7 10.1186/s12883-020-01845-7 PMC7333387 32620090

[7] Rocha-Filho PAS, Santos PV (2014) Headaches, quality of life, and academic performance in schoolchildren and adolescents. Headache 54:1194–1202. 10.1111/head.12394 10.1111/head.12394 24898739

[8] Headache. In: National Institute of Neurological Disorders and Stroke. https://www.ninds.nih.gov/healthinformation/disorders/headache. Accessed 7 June 2023

[9] Onofri A, Pensato U, Rosignoli C, et al (2023) Primary headache epidemiology in children and adolescents: a systematic review and meta-analysis. J Headache Pain 24:8. 10.1186/s10194-023-01541-0 10.1186/s10194-023-01541-0 PMC9926688 36782182

[10] Leonardi M, Grazzi L, D’Amico D, et al (2020) Global Burden of Headache Disorders in Children and Adolescents 2007-2017. Int J Environ Res Public Health 18:250. 10.3390/ijerph18010250 10.3390/ijerph18010250 PMC7795582 33396281

[11] Patniyot I, Qubty W (2023) Headache in Adolescents. Neurol Clin 41:177–192. 10.1016/j.ncl.2022.08.001 10.1016/j.ncl.2022.08.001 36400554

[12] GBD (2019) Diseases and Injuries Collaborators (2020) Global burden of 369 diseases and injuries in 204 countries and territories, 1990–2019: a systematic analysis for the Global Burden of Disease Study 2019. Lancet 396:1204–1222. 10.1016/S0140-6736(20)30925-9 10.1016/S0140-6736(20)30925-9 PMC7567026 33069326

[13] Gobel H The International Classification of Headache Disorders. In: ICHD-3. https://ichd-3.org/. Accessed 16 July 2023

[14] Global Burden of Disease: GBD cause and risk summaries. https://www.thelancet.com/gbd/summaries. Accessed 13 Jul 2023

[15] Joinpoint Regression Program. https://surveillance.cancer.gov/joinpoint/. Accessed 24 June 2023

[16] Dao JM, Qubty W (2018) Headache diagnosis in children and adolescents. Curr Pain Headache Rep 22:17. 10.1007/s11916-018-0675-7 10.1007/s11916-018-0675-7 29476266

[17] Xavier MKA, Pitangui ACR, Silva GRR et al (2015) Prevalence of headache in adolescents and association with use of computer and videogames. Ciênc saúde coletiva 20:3477–3486. 10.1590/1413-812320152011.19272014 10.1590/1413-812320152011.19272014 26602725

[18] Alberti A (2006) Headache and sleep. Sleep Med Rev 10:431–437. 10.1016/j.smrv.2006.03.003 10.1016/j.smrv.2006.03.003 16872851

[19] Rains JC, Poceta JS (2006) Headache and sleep disorders: review and clinical implications for headache management. Headache J Head Face Pain 46:1344–1363. 10.1111/j.1526-4610.2006.00578.x 10.1111/j.1526-4610.2006.00578.x 17040332

[20] Martin PR (2016) Stress and primary headache: review of the research and clinical management. Curr Pain Headache Rep 20:45. 10.1007/s11916-016-0576-6 10.1007/s11916-016-0576-6 27215628

[21] Reidy BL, Riddle EJ, Powers SW et al (2020) Clinic-based characterization of continuous headache in children and adolescents: comparing youth with chronic migraine to those with new daily persistent headache. Cephalalgia 40:1063–1069. 10.1177/0333102420920644 10.1177/0333102420920644 32336121

[22] Labbé EE, Murphy L, O’Brien C (1997) Psychosocial factors and prediction of headaches in college adults. Headache J Head Face Pain 37:1–5. 10.1046/j.1526-4610.1997.3701001.x 10.1046/j.1526-4610.1997.3701001.x 9046715

[23] Lederbogen F, Kirsch P, Haddad L et al (2011) City living and urban upbringing affect neural social stress processing in humans. Nature 474:498–501. 10.1038/nature10190 10.1038/nature10190 21697947

[24] Pacheco-Barrios K, Velasquez-Rimachi V, Navarro-Flores A et al (2023) Primary headache disorders in Latin America and the Caribbean: a meta-analysis of population-based studies. Cephalalgia 43:03331024221128265. 10.1177/03331024221128265 10.1177/03331024221128265 36606574

[25] Atun R, de Andrade LOM, Almeida G et al (2015) Health-system reform and universal health coverage in Latin America. Lancet 385:1230–1247. 10.1016/S0140-6736(14)61646-9 10.1016/S0140-6736(14)61646-9 25458725

[26] Li W, Bertisch SM, Mostofsky E et al (2019) Weather, ambient air pollution, and risk of migraine headache onset among patients with migraine. Environ Int 132:105100. 10.1016/j.envint.2019.105100 10.1016/j.envint.2019.105100 PMC7523027 31446321

[27] Riojas-Rodríguez H, da Silva AS, Texcalac-Sangrador JL, Moreno-Banda GL (2016). Air pollution management and control in Latin America and the Caribbean: implications for climate change. Rev Panam Salud Publica.

