# Supplementary material for: Rising trends in the burden of migraine and tension-type headache among adolescents and young adults globally, 1990 to 2019
Source: J Headache Pain. 2023 Jul 27;24(1):94. doi: 10.1186/s10194-023-01634-w (PMC10373384; doi:10.1186/s10194-023-01634-w)
Supplement: Supplementary file 1 — Additional file 1. [file 10194_2023_1634_MOESM1_ESM.pdf]

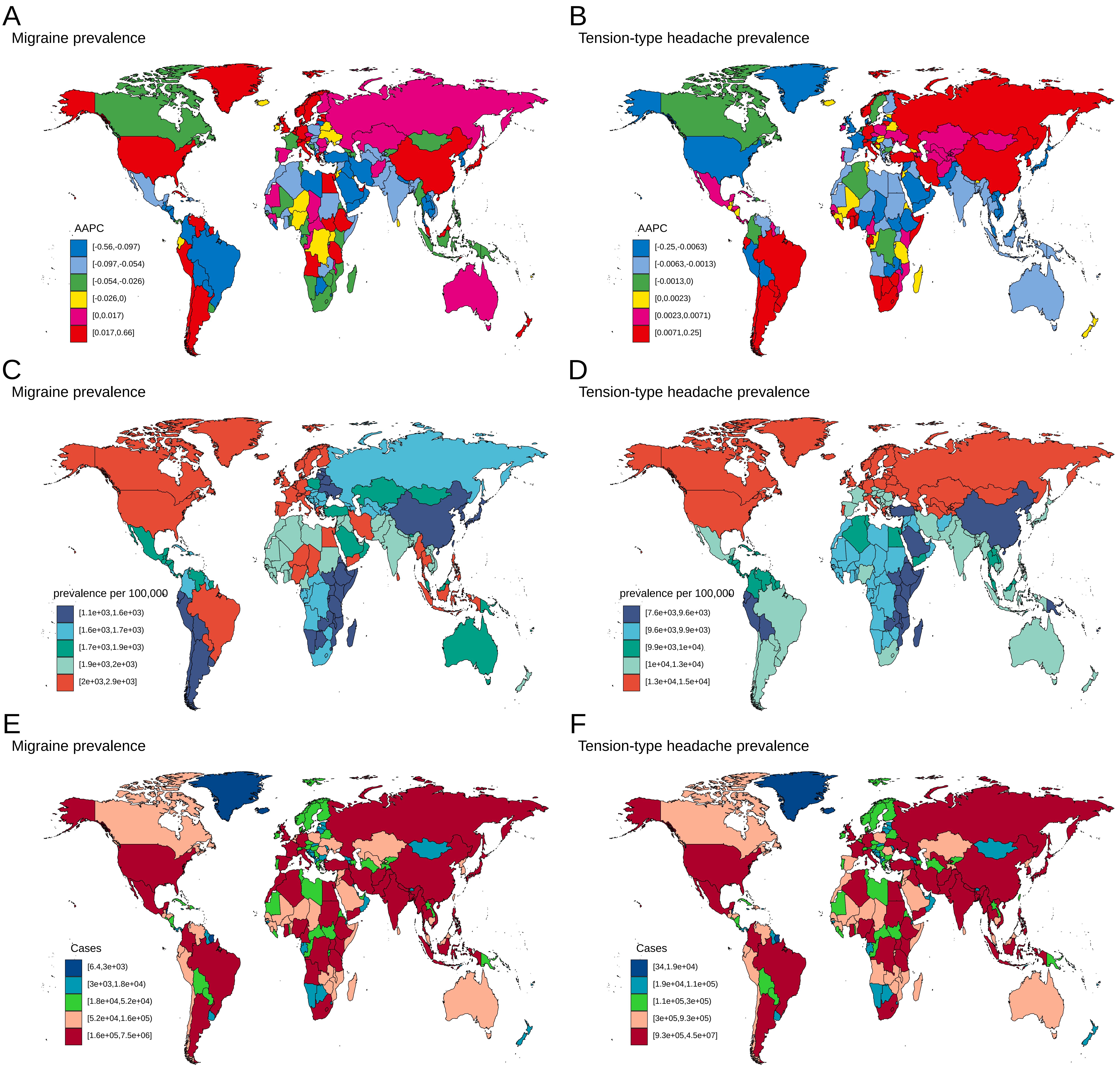
A
Migraine prevalence
AAPC
[-0.56,-0.097)
[-0.097,-0.054)
[-0.054,-0.026)
[-0.026,0)
[0,0.017)
[0.017,0.66]
B
Tension-type headache prevalence
AAPC
[-0.25,-0.0063)
[-0.0063,-0.0013)
[-0.0013,0)
[0,0.0023)
[0.0023,0.0071)
[0.0071,0.25]
C
Migraine prevalence
prevalence per 100,000
[1.1e+03,1.6e+03)
[1.6e+03,1.7e+03)
[1.7e+03,1.9e+03)
[1.9e+03,2e+03)
[2e+03,2.9e+03]
D
Tension-type headache prevalence
prevalence per 100,000
[7.6e+03,9.6e+03)
[9.6e+03,9.9e+03)
[9.9e+03,1e+04)
[1e+04,1.3e+04)
[1.3e+04,1.5e+04]
E
Migraine prevalence
Cases
[6.4,3e+03)
[3e+03,1.8e+04)
[1.8e+04,5.2e+04)
[5.2e+04,1.6e+05)
[1.6e+05,7.5e+06]
F
Tension-type headache prevalence
Cases
[34,1.9e+04)
[1.9e+04,1.1e+05)
[1.1e+05,3e+05)
[3e+05,9.3e+05)
[9.3e+05,4.5e+07]

Figure S1. Global map of AAPC for prevalence from 1990 to 2019 of Migraine (A) and tension-type headache (B), prevalence in 2019 of Migraine (C) and tension-type headache (D) and prevalence cases in 2019 of Migraine (E) and tension-type headache (F).

AAPC=average annual percentage change.

Figure S2. Global map of AAPC for DALYs from 1990 to 2019 of Migraine (A) and tension-type headache (B), DALYs in 2019 of Migraine (C) and tension-type headache (D) and DALYs cases in 2019 of Migraine (E) and tension-type headache (F).

AAPC=average annual percentage change.; DALYs=disability-adjusted life-years

Figure S1

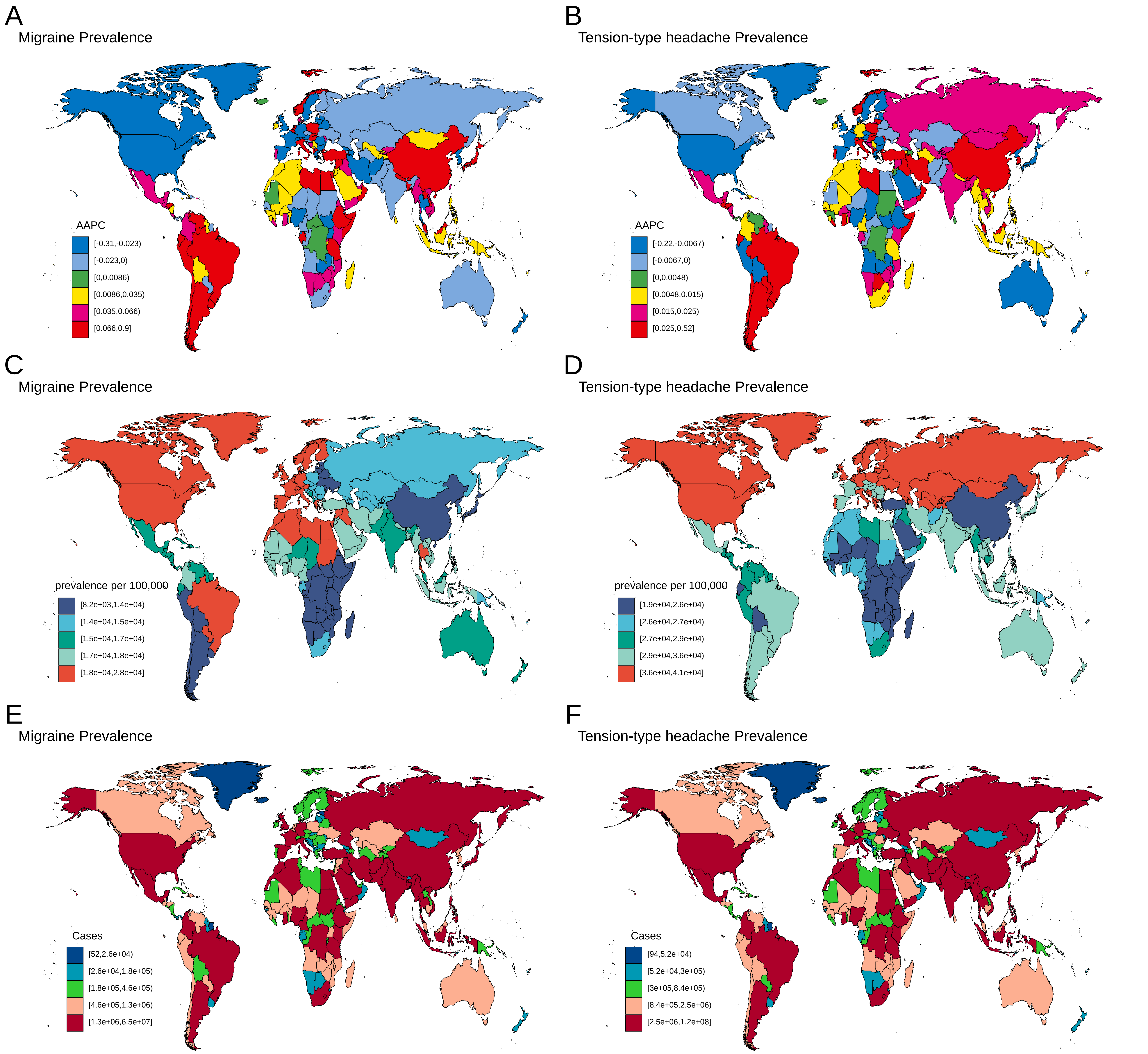
A
Migraine Prevalence
AAPC
[-0.31,-0.023)
[-0.023,0)
[0,0.0086)
[0.0086,0.035)
[0.035,0.066)
[0.066,0.9]
B
Tension-type headache Prevalence
AAPC
[-0.22,-0.0067)
[-0.0067,0)
[0,0.0048)
[0.0048,0.015)
[0.015,0.025)
[0.025,0.52]
C
Migraine Prevalence
prevalence per 100,000
[8.2e+03,1.4e+04)
[1.4e+04,1.5e+04)
[1.5e+04,1.7e+04)
[1.7e+04,1.8e+04)
[1.8e+04,2.8e+04]
D
Tension-type headache Prevalence
prevalence per 100,000
[1.9e+04,2.6e+04)
[2.6e+04,2.7e+04)
[2.7e+04,2.9e+04)
[2.9e+04,3.6e+04)
[3.6e+04,4.1e+04]
E
Migraine Prevalence
Cases
[52,2.6e+04)
[2.6e+04,1.8e+05)
[1.8e+05,4.6e+05)
[4.6e+05,1.3e+06)
[1.3e+06,6.5e+07]
F
Tension-type headache Prevalence
Cases
[94,5.2e+04)
[5.2e+04,3e+05)
[3e+05,8.4e+05)
[8.4e+05,2.5e+06)
[2.5e+06,1.2e+08]

Figure S2

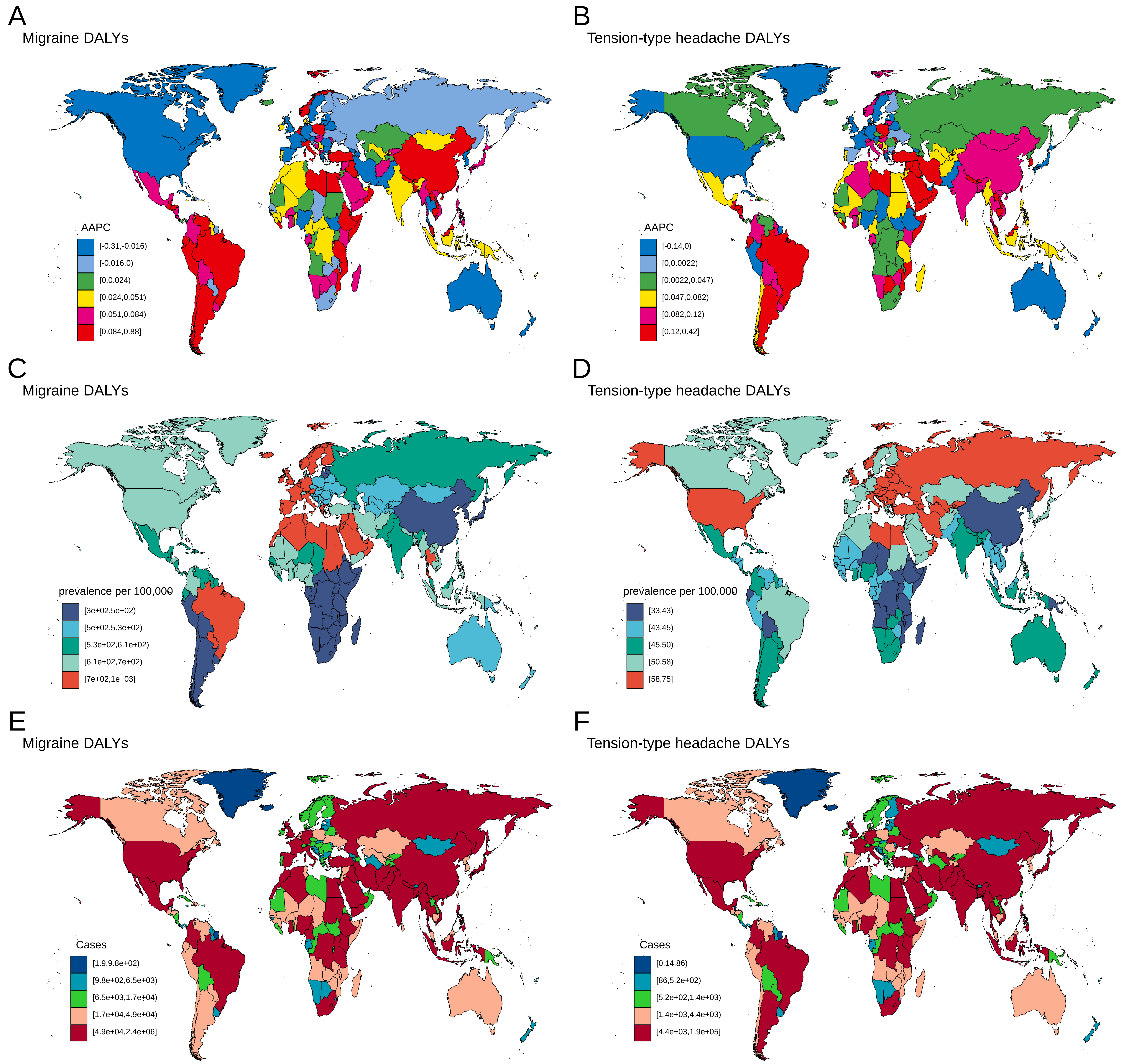
A
Migraine DALYs
AAPC
[-0.31,-0.016)
[-0.016,0)
[0,0.024)
[0.024,0.051)
[0.051,0.084)
[0.084,0.88]
B
Tension-type headache DALYs
AAPC
[-0.14,0)
[0,0.0022)
[0.0022,0.047)
[0.047,0.082)
[0.082,0.12)
[0.12,0.42]
C
Migraine DALYs
prevalence per 100,000
[3e+02,5e+02)
[5e+02,5.3e+02)
[5.3e+02,6.1e+02)
[6.1e+02,7e+02)
[7e+02,1e+03]
D
Tension-type headache DALYs
prevalence per 100,000
[33,43)
[43,45)
[45,50)
[50,58)
[58,75]
E
Migraine DALYs
Cases
[1.9,9.8e+02)
[9.8e+02,6.5e+03)
[6.5e+03,1.7e+04)
[1.7e+04,4.9e+04)
[4.9e+04,2.4e+06]
F
Tension-type headache DALYs
Cases
[0.14,86)
[86,5.2e+02)
[5.2e+02,1.4e+03)
[1.4e+03,4.4e+03)
[4.4e+03,1.9e+05]
